# Asynaptic feature and heterogeneous distribution of the cholinergic innervation of the globus pallidus in primates

**DOI:** 10.1007/s00429-014-0960-0

**Published:** 2014-12-19

**Authors:** Lara Eid, André Parent, Martin Parent

**Affiliations:** 1 Centre de recherche de l’Institut universitaire en santé mentale de Québec (CRIUSMQ), F-6530-1, 2601, ch. de la Canardière, Quebec, QC G1J 2G3 Canada; 2 Department of Psychiatry and Neuroscience, Faculty of Medicine, Université Laval, Quebec, QC G1V 0A6 Canada

**Keywords:** Acetylcholine, Basal ganglia, ChAT immunohistochemistry, Monkey, Pallidum, Stereology

## Abstract

The internal (GPi) and external (GPe) segments of the primate globus pallidus receive a significant cholinergic (ACh) innervation from the brainstem pedunculopontine tegmental nucleus. The present immunohistochemical study describes this innervation in the squirrel monkey (*Saimiri sciureus*), as visualized with an antibody raised against choline acetyltransferase (ChAT). At the light microscopic level, unbiased stereological quantification of ChAT positive (+) axon varicosities reveals a significantly lower density of innervation in GPi (0.26 ± 0.03 × 10^6^) than in GPe (0.47 ± 0.07 × 10^6^ varicosities/mm^3^ of tissue), with the anterior half of both segments more densely innervated than the posterior half. Neuronal density of GPi (3.00 ± 0.13 × 10^3^ neurons/mm^3^) and GPe (3.62 ± 0.22 × 10^3^ neurons/mm^3^) yields a mean ratio of ChAT+ axon varicosities per pallidal neuron of 74 ± 10 in the GPi and 128 ± 28 in the GPe. At the electron microscopic level, the pallidal ChAT+ axon varicosities are significantly smaller than their unlabeled counterparts, but are comparable in size and shape in the two pallidal segments. Only a minority of ChAT+ varicosities displays a synaptic specialization (12 % in the GPi and 17 % in the GPe); these scarce synaptic contacts are mostly of the symmetrical type and occur exclusively on pallidal dendrites. No ChAT+ axo-axonic synaptic contacts are observed, suggesting that ACh exerts its modulatory action on pallidal afferents through diffuse transmission, whereas pallidal neurons may be influenced by both volumic and synaptic delivery of ACh.

## Introduction

Acetylcholine (ACh) is involved in a wide variety of central nervous system functions, including neuronal plasticity, attention, learning and memory (Hasselmo 1995; Dykes 1997; Baxter and Chiba 1999; Sarter et al. 2003). It is known to be implicated in the activity of the various basal ganglia components, particularly the striatum, which is one of the most densely ACh innervated structures of the entire brain (Mesulam et al. 1992; Contant et al. 1996; Gold 2003; Power et al. 2003; Bonsi et al. 2011; Goldberg et al. 2012; Avena and Rada 2012). This massive ACh innervation arises essentially from a relatively small population of large, aspiny interneurons (McGeer et al. 1971; Woolf and Butcher 1981; Bolam et al. 1984; Woolf 1991), with a minor contribution from brainstem (Dautan et al. 2014) and basal forebrain (Mesulam et al. 1992) neurons. By comparison, the ACh innervation of the internal (GPi) and external (GPe) segments of the globus pallidus is much less dense and originates principally from the pedunculopontine tegmental nucleus (PPN) located in the mesopontine brainstem tegmentum (DeVito et al. 1980; Woolf and Butcher 1986; Mesulam et al. 1992; Sutoo et al. 1994; Charara and Parent 1994).

Pallidal neurons in monkeys are known to display muscarinic receptors of the M1 and M2 subtypes and, to a lesser degree, nicotinic receptors (Miyoshi et al. 1989; Smiley et al. 1999; Quik et al. 2000). The expression of such a wide variety of ACh receptors may explain why the pharmacological application of ACh upon pallidal neurons in both rodents and primates can either increase or decrease their neuronal firing rates (Lénárd et al. 1995). Receptor heterogeneity could also account for the fact that the electrical stimulation of the PPN produces both excitatory and inhibitory effects upon neurons of the rat entopeduncular nucleus, the rodent homologue of the primate GPi (Scarnati et al. 1988).

The regional ACh innervation of the various basal ganglia components has been studied in some details in human by means of ChAT immunohistochemistry (Mesulam et al. 1992; Sutoo et al. 1994). These investigations indicate that the ACh innervation of the human globus pallidus is less dense than that of the striatum and subthalamic nucleus, but higher than that of the substantia nigra pars compacta (Mesulam et al. 1992; Sutoo et al. 1994). Furthermore, the ACh innervation of the human pallidum was found to be denser than its dopaminergic counterpart (Sutoo et al. 1994), but no detailed morphological and quantitative information on the ACh innervation of the primate pallidum is currently available. At the ultrastructural level, examination of various central nervous system structures in rats, cats and monkeys have demonstrated that the ACh innervation is predominantly of the asynaptic type (for reviews see Descarries and Mechawar 2000; Descarries and Parent 2014). Ultrastructural evidence for the presence of pallidal ACh axon varicosities have been gathered following examination of the rat entopeduncular nucleus (Clarke et al. 1996, 1997), but similar data for the primate pallidum are unavailable. This lack of information regarding the ACh innervation of primate pallidum has led us to initiate a quantitative study of the regional distribution of pallidal ACh innervation, as well as a detailed immunoelectron microscopic study of the intrinsic and relational features of the ACh terminals in the pallidum of squirrel monkeys. The present study is part of a larger research program, whose aim is to provide a detailed picture of morphological substratum whereby the various chemospecific brainstem afferents (serotoninergic, cholinergic and dopaminergic) influence the activity of pallidal neurons in primates. Here, we compare the ACh innervation of the squirrel monkey GPi and GPe, which, despite their morphological similarity, are involved in quite different aspects of basal ganglia functions. Being reciprocally linked with the subthalamic nucleus, the GPe forms one of the major ancillary loops that are so characteristic of basal ganglia circuitry (Parent and Hazrati 1995a). In contrast, the GPi, whose neurons are endowed with a markedly collateralized and widely distributed axon (Parent et al. 2001), is considered as one of the major output structures of the basal ganglia (Parent and Hazrati 1995b). We hope that such a detailed comparison of the ACh innervation of these two functionally distinct components of the squirrel monkey’s pallidum will further our understanding of the role that this neurotransmitter plays in the functional organization of primate basal ganglia.

## Materials and methods

### Animals

This study was carried out on six young (3–4 years old) adult male squirrel monkeys (*Saimiri sciureus*, Buckshire Corporation, Perkasie, PA, USA) weighing 895 ± 17 g. Animals were housed under a 12 h light–dark cycle, with food and water ad libitum. Our experimental protocol was approved by the ‘Comité de Protection des Animaux de l’Université Laval’, and all procedures involving animals and their care were made in accordance with the Canadian Council on Animal Care’s Guide to the Care and Use of Experimental Animals (Ed2). Maximum efforts were made to minimize the number of animals used.

### Tissue processing

Animals were first deeply anesthetized with a mixture of ketamine (20 mg/kg, i.m.) and xylazine (4 mg/kg, i.m.), along with acepromazine (0.5 mg/kg, i.m.). They were then perfused transcardially with 200 mL of ice-cold sodium phosphate-buffered saline (PBS, 50 mM; pH 7.4), followed by 500 mL of 3.0 % acrolein in phosphate buffer (PB, 50 mM; pH 7.4) and by 1 L of cold 4 % paraformaldehyde (PFA). Brains were rapidly dissected out, postfixed by immersion in 4 % PFA for 1 h at 4 °C and cut with a vibratome (Leica) into 50 µm-thick sections collected in PBS (100 mM; pH 7.4). Brains were cut along the coronal plane, except for the left hemisphere of one monkey that was cut along the sagittal plane and the left hemisphere of another monkey that was cryoprotected in a 30 % sucrose solution for 3 days and cut in 50 µm-thick horizontal sections using a freezing microtome. Sagittal, horizontal and coronal sections were used to describe the ascending ACh pathways from the PPN, whereas stereological and electron microscopical analyses were exclusively conducted on coronal sections.

### Immunohistochemistry

The polyclonal antibody against the rate-limiting enzyme for ACh synthesis, choline acetyltransferase (ChAT; catalog no. AB144P, EMD Millipore Corporation, Billerica, MA, USA), was raised in goat against the human placental enzyme. It was affinity-purified and characterized by western blot in brain tissue. The density and arrangement of axonal arborizations visualized on brain sections stained with this particular antibody was typical of ACh neurons, as it corresponds exactly to the staining features obtained with other anti-ChAT sera (Contant et al. 1996; Mechawar et al. 2000). Furthermore, the immunostaining pattern of cell bodies obtained with this antibody also perfectly matched the distribution of neurons expressing ChAT mRNA, as detected by in situ hybridization (Wang and Morales 2009). The processing of monkey brain tissue without primary or secondary antibody completely abolishes the immunostaining.

In preparation for *light microscopy*, free-floating brain sections from all 6 monkeys were sequentially incubated at room temperature (RT), unless stated otherwise, in (1) a solution of 0.3 % hydrogen peroxide and 50 % ethanol for 30 min to eliminate endogenous peroxidase activity, (2) a solution of 0.5 % NaBH_4_ diluted in PBS (30 min), both followed by several rinses in PBS, (3) a blocking solution of PBS, containing 2 % normal rabbit serum and 2 % Triton X-100 (1 h), (4) the same solution to which a 1:25 dilution of goat polyclonal antibody against ChAT was added (48 h), followed by several rinses in PBS, and (5) the same blocking solution containing a 1:1,000 dilution of biotinylated rabbit anti-goat antibody (catalog no. BA5000, Vector Laboratories, Burlingame, CA, USA; 1 h). After rinses in PBS, sections were incubated for 1 h in avidin–biotin-peroxidase complex (catalog no. PK-4000, Vector Laboratories) diluted 1:100 in PBS. They were then rinsed twice in PBS and once in Tris–saline buffer (TBS; 50 mM, pH 7.4), and the bound peroxidase was revealed by incubating the sections for 2 min in a 0.05 % solution of 3,3′diaminobenzidine (catalog no. D5637; Sigma, St-Louis, MO, USA) in TBS to which 0.005 % H_2_O_2_ was added. Several rinses in TBS followed by PB stopped the reaction, and sections were mounted on gelatin-coated slides, air-dried overnight, dehydrated in graded alcohol series, cleared in toluene and coverslipped with Permount.

In preparation for *electron microscopy*, sections from four of the six monkeys were incubated with the same primary and secondary antibodies as described above, but without Triton X-100, which was replaced by 0.5 % cold fish gelatin. These sections did not undergo the first step in peroxide-ethanol. Primary antibody was incubated 24 h at RT and then 24 h at 4 °C. Secondary antibody was diluted 1:500 in blocking solution and incubated for 1.5 h. Sections were then osmicated, dehydrated in ethanol and propylene oxide and flat-embedded in Durcupan (catalog no. 44611-14; Fluka, Buchs, Switzerland) to be processed and examined as described below.

In preparation for *immunofluorescence*, 4 coronal sections from one monkey were processed for ChAT immunohistochemistry at the level of the anterior, mid-portion and posterior GPi and at the mid portion of the PPN. After several washes in PBS, free-floating sections were incubated in a solution of 0.5 % NaBH_4_ diluted in PBS followed by several rinses in PBS. They were then incubated according to the sequence outlined above for light microscopy preparation, except that the biotinylated secondary antibody was incubated for 2 h, followed by rinses in PBS and an incubation in a 1: 200 solution of Alexa Fluor 488 streptavidin (catalogue no. S11223; Molecular Probes, Life Technologies Corporation, Burlington, ON, Canada) diluted in blocking solution for 2 h. Sections were rinsed in PBS and mounted on gelatin-coated slides, air-dried overnight and processed with autofluorescence eliminator reagent (catalog no. 2160, Millipore, Temecula, CA, USA), according to instructions from the manufacturer. They were then coverslipped with DAKO fluorescent mounting medium (catalog no. S3023, Dako North America, Carpinteria, CA, USA).

### Section intervals used for the description of ACh ascending pathways

The trajectories of the ACh ascending pathways originating from the PPN were visualized on sagittal, horizontal and coronal sections that were processed for ChAT immunostaining. To provide a faithful description of the initial trajectory, selected sections through the brainstem were taken at intervals of 150 µm, whereas other sections, used to study the morphological characteristics and distribution patterns of ACh axon varicosities and neuronal somata, were separated by 300 µm. Pathways were delineated with the help of a light microscope equipped with a 20×/0.70 objective. Sections processed for ChAT immunofluorescence, as described above, were imaged with a LSM 700 confocal microscope (Zeiss Canada) equipped with four solid-state lasers and an EC Plan neofluar 20×/0.5 objective.

### Quantitative assessment of the density of ChAT-immunoreactive axon terminals

The stereological procedures used in the present study have been described in details elsewhere (Eid et al. 2013). In brief, the number of ChAT-immunoreactive axon terminals in the GPi and GPe was assessed on coronal sections examined at the light microscopic level using an unbiased stereological approach and the StereoInvestigator software (v.10.54, MicroBrightField, Colchester, VT, USA). Eight equally spaced transverse sections were selected across the entire rostrocaudal extent of both pallidal segments in each of the six monkeys. The GPi being smaller than the GPe, the interval between each section had to be smaller (300 µm) for the GPi than for the GPe (600 µm) in order to obtain 8 equally spaced sections for each pallidal segment. The first section was always selected at random and other sections were chosen according to the interval mentioned above. Each pallidal segment was then divided into eight sectors, enabling a more precise description of the regional distribution of ChAT positive (+) axon varicosities throughout the GPi and GPe. To do so, the contour of the GPi and GPe was first outlined at a low magnification on each coronal section. Then, two lines, forming an acute angle, were traced; one passing through the dorsal aspect of the lenticular nucleus and the other through its ventral aspect. A third line was traced as a bisector, dividing each pallidal segment into ventral and dorsal sectors. Another line, perpendicular to and centered on the bisector was traced to delineate four sectors on each pallidal segment and each brain section. The anteroposterior axis was then divided in two by considering the first four transverse sections as representative of anterior sectors and the last four of posterior sectors (see Eid et al. 2013 for details). The sampling of ChAT+ axon varicosities began by randomly placing a grid formed by 400 × 400 µm squares over each section. At each intersection of the grid that fell into the sector, a 30 × 30 µm counting frame was drawn and examined with a 100×/1.30 oil-immersion objective. ChAT+ axon varicosities appear under light microscope as round or ovoid axonal dilation measuring >0.25 µm in transverse diameter. Varicosities were counted whenever one such profile was encountered inside the counting frame, did not touch the exclusion line and came into focus inside a 10 µm-thick optical disector centered in the section. For each counting frame, the thickness of the mounted tissue was measured, yielding to mean values of 20.6 ± 0.7 µm in the GPi and 20.8 ± 0.8 µm in the GPe, providing a lower and upper guard zone of approximately 5 µm. An average of 232 ± 69 axon varicosities were counted in each sector of the GPi and GPe, yielding to coefficients of error (Gunderson, *m* = 1 and second Schmitz-Hof) ranging between 0.03 and 0.18. For each sector, the density of ChAT innervation was expressed in 10^6^ axon varicosities per mm^3^ of tissue, using the total number of axon varicosities calculated by the optical disector and the volume of the sector estimated by Cavalieri’s method. The density of ChAT+ axon varicosities in the entire GPi and GPe was estimated by using the same approach. Overall, the stereological procedures used in the present study, including section intervals, grids and counting frame sizes, were exactly the same as those used in our previous investigation of the serotonin (5-HT) innervation of the monkey pallidum (Eid et al. 2013), allowing a direct comparison between the 5-HT and ACh pallidal innervations.

### Quantitative assessment of the neuronal population in the GPi and GPe

Adjacent coronal sections across the GPi and GPe of each monkey were Nissl-stained and served to estimate the total neuronal population in each pallidal segment according to the method used by Eid et al. (2013). In brief, sections were mounted on gelatin-coated slides, air-dried overnight, dehydrated in 70 % ethanol for 10 min, rehydrated in distilled water for 5 min and stained with cresyl violet for 20 min. Sections were then dehydrated in graded alcohol series, cleared in toluene and coverslipped with Permount. In each pallidal segment, eight sectors were delineated as described above and sampled by randomly placing a grid formed by 360 × 360 µm squares over each section. At each intersection of the grid that fell into the sector, a 200 × 200 µm counting frame was drawn and examined with a 20×/0.70 objective. Pallidal neurons being uniformly voluminous, they could easily be differentiated from the small glial cells and were counted when the nucleolus came into focus inside a 12 µm-thick optical disector centered in the section, yielding upper and lower guard zone of approximately 4 µm. Neuronal population density was expressed in 10^3^ neurons per mm^3^ of tissue, using the total number of neurons calculated by the optical disector and the volumes estimated by Cavalieri’s method.

### Quantitative assessment of the ACh neuronal population in the GPe

The same eight sections per monkey that served to estimate the number of ChAT+ axon varicosities in the GPi and GPe were also used to estimate the number of ChAT+ cell bodies detected in the GPe. We used stereological parameters that were exactly the same as those described above for the estimation of pallidal neuronal populations from Nissl-stained adjacent sections.

### Ultrastructural features of ChAT-immunoreactive axon varicosities

For each of the four monkeys that were used for electron microscopy, quadrangular pieces were cut in the center of GPi and GPe from two flat-embedded ChAT-immunostained sections taken at the mid anteroposterior level of the pallidum (AP = 10 mm, according to the stereotaxic atlas of Emmers and Akert (1962)). The quadrangular pieces were glued on the tip of a resin block and cut with an ultramicrotome (Leica EM UC7) in ultrathin sections (~80 nm), which were collected on bare 150-mesh copper grids and stained with lead citrate. Grids were examined with a *Tecnai 12* transmission electron microscope (100 kV; Philips Electronic) equipped with an integrated Mega-View II digital camera (SIS, Germany). Profiles of varicosities were identified by their diameter >0.25 µm and their content in synaptic vesicles, often associated with one or more mitochondria. The ChAT+ axon varicosities were randomly sampled at a working magnification of 11,500× by taking a picture every time such profile was encountered, until 40 or more pictures were available for analysis in each pallidal segment of each animal.

The public domain *ImageJ* processing software (NIH; v.1.45) was used for the analysis of ChAT+ and unlabeled axon varicosities randomly selected from the same pictures and for which the long and short axes, as well as cross-sectional area were measured. Each varicosity was then categorized as containing or not a mitochondrion, and as showing or not a synaptic junctional complex, i.e. a localized straightening of apposed plasma membranes accompanied by a slight widening of the intercellular space and a thickening of the pre- and/or postsynaptic membrane. For each synaptic junction, the length of junctional complex was measured and the target identified, and each was categorized as symmetrical or asymmetrical. The synaptic incidence observed in single section was then extrapolated to whole volume of varicosities by means of the formula of Beaudet and Sotelo (1981), using the long axis as diameter according to Umbriaco et al. (1994). This formula allows the prediction of seeing a synapse if there is one on every varicosity by taking into account the average size of varicosity profiles, the length of their junctional complexes, and the thickness of the section. The synaptic incidence is inferred by comparison to this predicted value. This procedure has been validated experimentally by Umbriaco et al. (1994) who found almost identical values of synaptic incidence for a large population of ChAT+ cortical varicosities in serial sections across their entire volume, and a randomized single section sample of these same varicosities. The microenvironment surrounding ten ChAT+ axon varicosities per pallidal segments of the four monkeys used for electron microscopy was examined. Unlabeled profiles directly apposed to immunostained axon varicosities were identified as unmyelinated or myelinated axon, cell body, dendrite or astrocyte and measured for long and short axes. The proportion for each type of profile surrounding the ChAT+ axon varicosities was then calculated.

### Statistics

Wilcoxon-signed rank test was used to assess differences in the density of ChAT+ axon varicosities between the anterior and posterior halves of each pallidal segment. The same statistical approach was employed to detect differences between the lateral and medial halves and between the dorsal and ventral halves, as well as between the entire GPi and GPe. Statistical differences in neuronal densities between the anterior, posterior, dorsal, ventral, lateral and medial pallidal sectors, as well as between the two pallidal segments were assessed using the same approach. One-way ANOVA followed by Tukey’s multiple comparison tests was used to identify differences in dimensions and synaptic incidence between ChAT+ and unlabeled axon varicosities, as well as between ChAT+ varicosities present in the GPi and those of the GPe. Differences were considered significant at *P* < 0.05. Statistical analysis was done using GraphPad Prism software (v. 5.0; GraphPad Software, San Diego, CA, USA) and SPSS (v. 20.0; IBM Corp., Armonk, NY, USA). Mean and standard error of the mean are used throughout the text as central tendency and dispersion measure, respectively.

## Results

### ChAT-immunoreactive ascending pathways

**Fig. 1:**
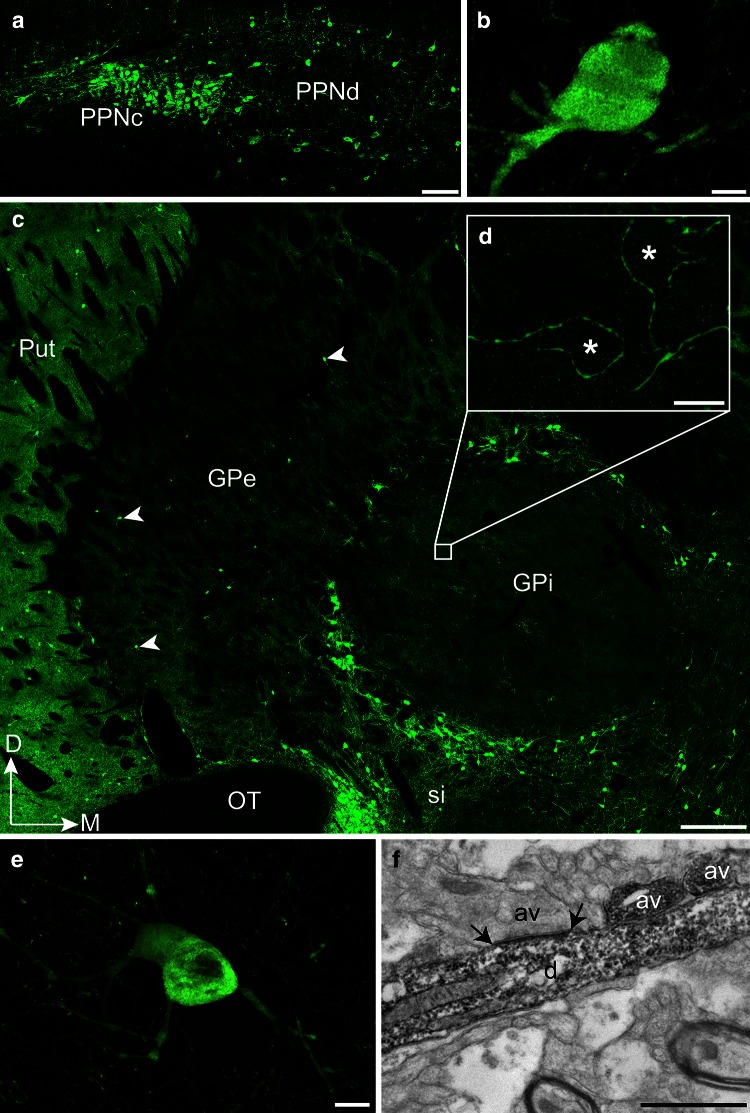
Coronal sections through the pedunculopontine tegmental nucleus (**a**, **b**) and the globus pallidus (**c**–**e**) of the squirrel monkey showing ChAT-labeled elements visualized with the confocal (**a**–**e**) and electron microscope (**f**). **a** Difference between the pars compacta (PPNc) and the pars dissipata (PPNd) of the pedunculopontine nucleus in regards to the number and density of ChAT+ neurons. **b** Higher magnification of a ChAT+ neuron located in the PPNc, with its typical round-ovoid cell body from which only a few thick primary dendrites emerge (10 µm-thick z-stack). **c** Low power view of the pallidal complex taken at mid anteroposterior level. Note the marked difference in the intensity of ChAT immunostaining between the pallidum and the putamen. The ChAT+ neurons in the internal medullary lamina, which forms a fluorescent neuronal ring around the GPi, are clearly visible, as well as some of the ChAT+ neurons present within the core of the GPe (*arrowheads*). **d** Enlargement of the typical pericellular arrangement of ChAT+ axons observed in the GPi (18 µm-thick z-stack). **e** Higher magnification of a ChAT+ cell body observed in the GPe. Its exact location is indicated by the lowest arrowhead in the GPe in **c** (2.4 µm-thick z-stack). The cell bodies that belong to intrinsic ChAT+ pallidal neurons are smaller than those that populate the nucleus basalis of Meynert or the internal medullary lamina. They are endowed with 2–4 thick primary dendrites. **f** Example of a ChAT+ dendrite observed in the dorsolateral sector of the GPe, as visualized by electron microscopy. Note that this longitudinally cut ChAT+ dendrite receives an asymmetrical synaptic contact (between *arrows*) from an unlabeled axon varicosity (av). Also note two ChAT+ axon varicosities (av) that are juxtaposed to the ChAT+ dendrite. *Scale bars* 300 µm in **a**, 500 µm in **c**, 10 µm in **b**, **d** and **e** and 1 µm in **f**

**Fig. 2:**
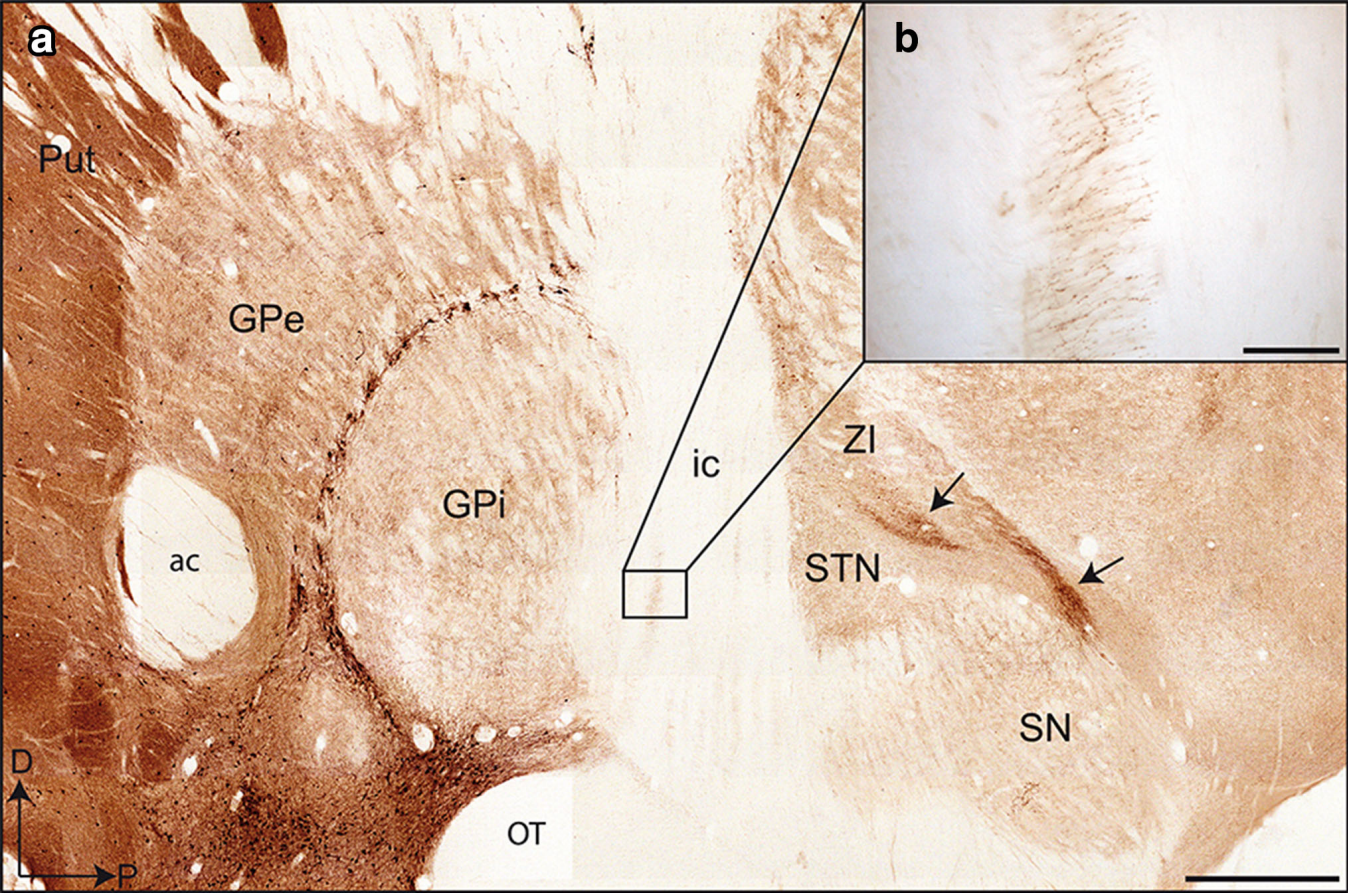
**a** Photomicrograph of ACh pathways as seen on a sagittal section taken at the mid mediolateral pallidal level. The ChAT+ axon fibers segregate into several distinct bundles, some running along the dorsal surface of the substantia nigra (SN), others surrounding the subthalamic nucleus (STN) and still others circumventing the zona incerta (ZI, *arrows*). **b** Higher magnification of ChAT+ fibers that pierce the internal capsule to invade the ventral part of the GPi through its caudal extent. *Scale bars* 1 mm in **a** and 100 µm in **b**

The ACh innervation of the globus pallidus in monkeys originates mostly from the brainstem PPN, which comprises two distinct portions termed *pars compacta* (PPNc) and *pars dissipata* (PPNd) (Fig. 1a). The PPNc of the squirrel monkey harbours numerous, densely packed, round or ovoid ChAT+ cell bodies giving rise to only a few thick primary dendrites (Fig. 1b). Morphologically similar ChAT+ neurons occur in the PPNd, but these neurons are less numerous and more widely distributed than those in the PPNc. Overall, the density of ChAT+ neurons in the two parts of the PPN is lower than in the laterodorsal tegmental nucleus (LTD), a ACh brainstem nucleus that is known to innervate massively the thalamus (Sofroniew et al. 1985; Woolf and Butcher 1986; Hallanger et al. 1987; Steriade et al. 1988; Paré et al. 1988). A careful examination of coronal, sagittal and horizontal sections immunostained for ChAT has revealed that the labeled axons originating from ChAT+ neurons in the PPN form a diffuse bundle that ascends within the midbrain tegmentum. Most of these ChAT+ axons follow the cerebellar peduncle decussation and segregate into several distinct dense bundles, some running along the dorsomedial surface of the substantia nigra, others surrounding the subthalamic nucleus and still others circumventing the zona incerta, while coursing along the lenticular and thalamic fasciculi (Fig. 2a). At the caudal pallidal level, ChAT+ fibers present in the lenticular fasciculus pierce the internal capsule to enter the pallidum (Fig. 2b). At mid pallidal level, numerous fibers course in the ventral portion of the internal capsule before invading the ansa lenticularis. Rostrally, the ChAT+ ascending fibers innervate the pallidal complex by entering through the ventromedial edge of the globus pallidus.

### ChAT-immunoreactive neurons within the confines of the pallidum

The pallidal complex in primates lies immediately above an impressive collection of large (43 ± 10 µm) and multipolar ChAT+ neurons that are scattered within the substantia innominata and form the essential neuronal component of the so-called nucleus basalis of Meynert. Despite their close proximity to the pallidum, the ChAT+ neurons of the nucleus basalis remain largely segregated from the smaller ChAT negative (−) pallidal neurons. Although some large ChAT+ neurons morphologically similar and topographically related to neurons of nucleus basalis do invade the internal medullary lamina that separates the GPi from the GPe, the former pallidal segment remains largely devoid of ChAT+ cell bodies (Fig. 1c). In contrast, a small number of ChAT+ nerve cell bodies are scattered within the core of the GPe (Fig. 1c, e). The cell body of the ChAT+ neurons in the GPe is smaller (26 ± 4 µm) than that of the immunoreactive neurons present in the internal medullary lamina (51 ± 6 µm), and gives rise to 2–4 thick primary dendrites (Fig. 1e). The quantitative stereological analysis of these ChAT+ pallidal neurons indicates that their density is of 55 ± 6 neurons/mm^3^, compared to 3,000 ± 127 pallidal neurons/mm^3^, so that they appear to represent not more than 1.8 % of the total GPe neuronal population. These ChAT+ GPe neurons are uniformly distributed along the rostrocaudal and mediolateral axes, but are more than twice as abundant (*P* = 0.001) in the ventral half of the GPe (86 ± 6 neurons/mm^3^) than in the dorsal half (38 ± 3 neurons/mm^3^).

### ChAT-immunoreactive axons in the pallidal complex

**Fig. 3:**
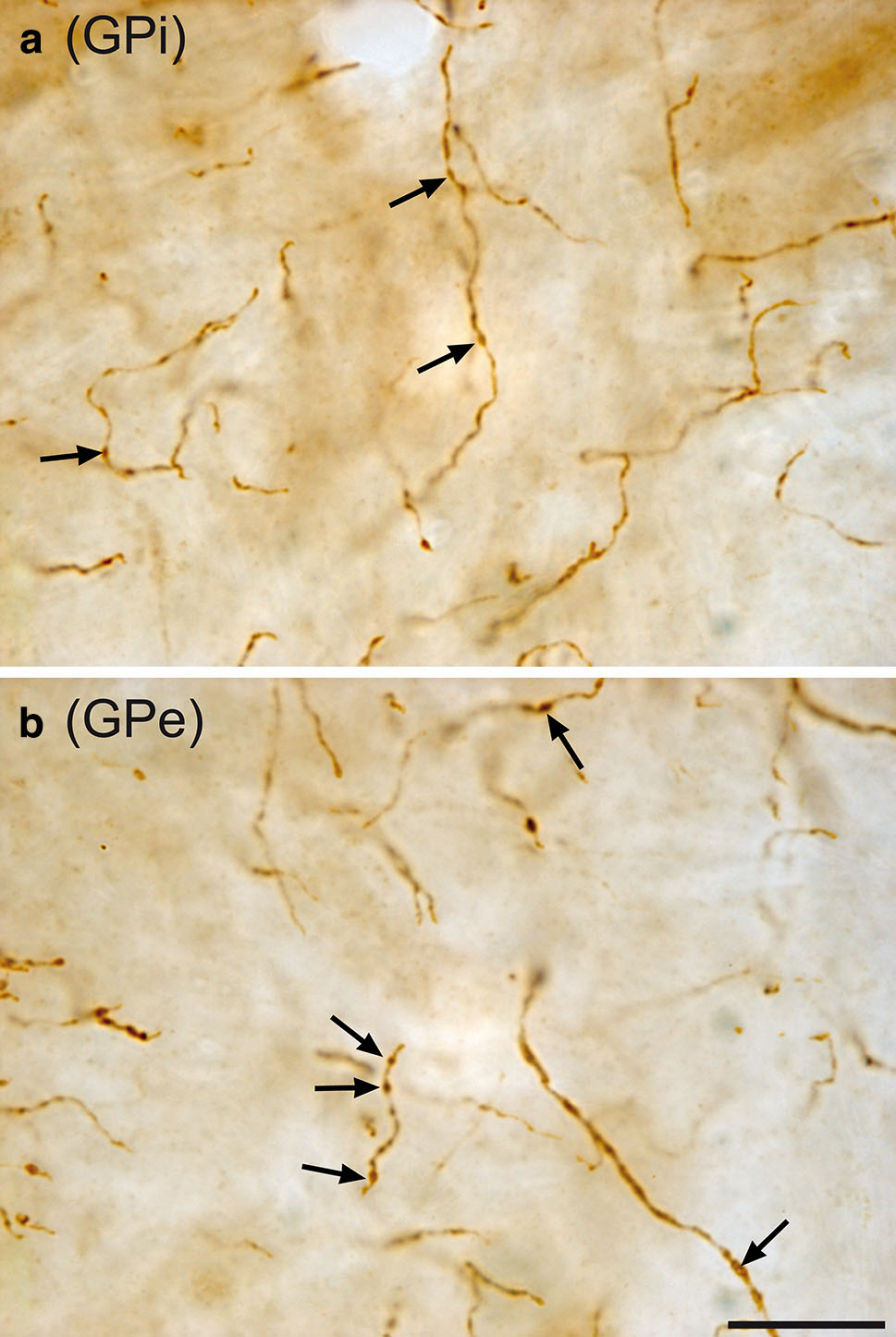
High magnification of ChAT+ axons in the GPi (**a**) and the GPe (**b**), with *black arrows* pointing to axon varicosities that could be easily delineated and counted using unbiased stereological approach. *Scale bar* 20 µm

Overall, the intensity of ChAT immunostaining in the GPi and GPe is significantly lower than that of the adjoining putamen, whose ACh innervation derives chiefly from a small population of giant local circuit neurons (Fig. 1c). At mid anteroposterior levels of the pallidum, the ChAT immunoreactivity is slightly more intense in GPi than in GPe, but regional variations in staining intensity exist throughout the entire rostrocaudal extent of the pallidal complex. The examination of immunoperoxidase-stained sections reveals the presence of many thin and varicose ChAT+ fibers in both GPi and GPe (Fig. 3). In the GPi, these fibers are thin and form a delicate network in which typical pericellular arrangements are often found, as seen under the confocal microscope (Fig. 1d). In the GPe, the labeled fibers are more uniformly distributed and slightly thicker, and some of them might represent fibers of passage. The number of ChAT+ fibers is roughly similar in the GPi and GPe, but the labeled fibers in the GPi tend to be less varicose than those of the GPe. When examined at a high magnification, the ChAT+ axon varicosities appear similar in shape and size in the two pallidal segments; these elements can be easily delineated and counted on such immunostained preparations (Fig. 3).

### Density of ChAT-immunoreactive axon varicosities in the two pallidal segments

**Fig. 4:**
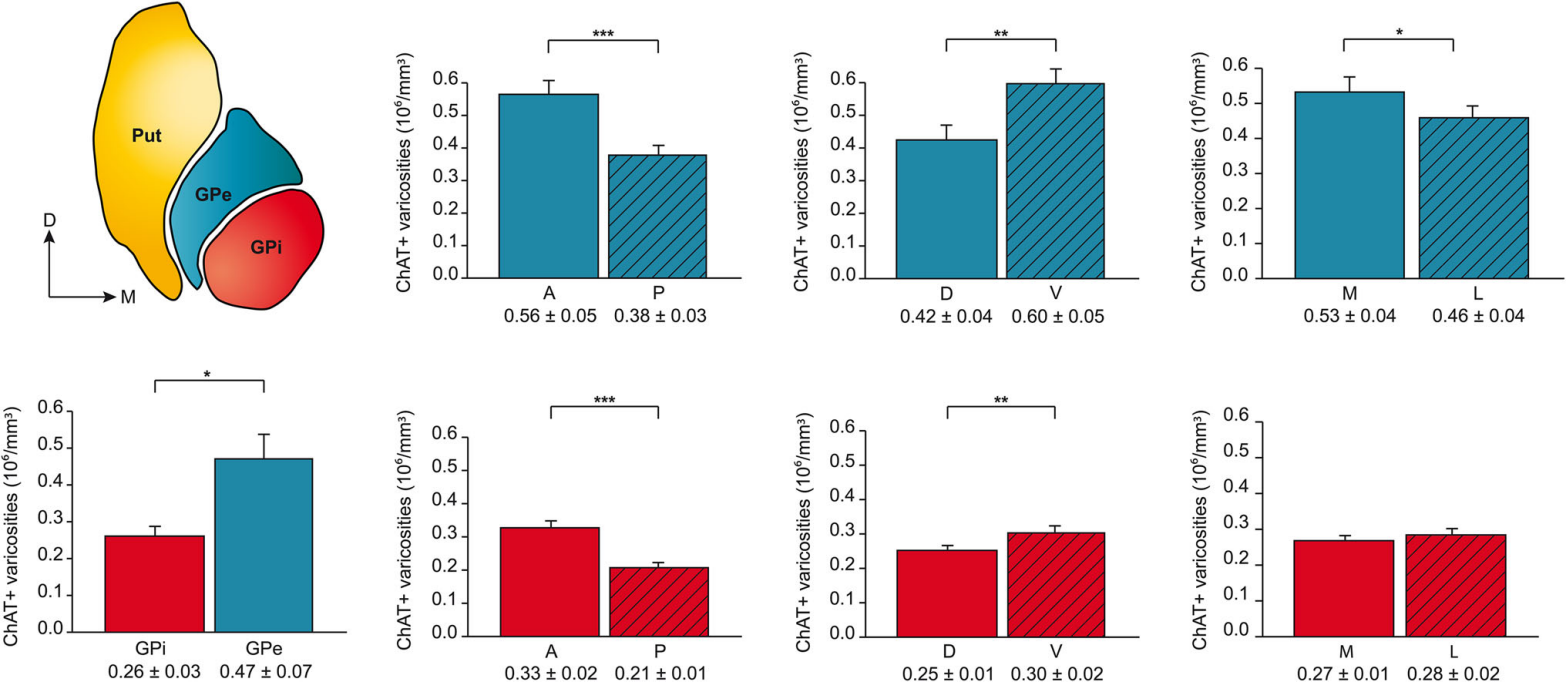
*Histograms* indicating, on the *bottom left*, the average densities of ChAT+ axon varicosities for the whole GPi (*red*) and GPe (*blue*) and, on the right, the average densities of ChAT+ axon varicosities for the anterior (A) and posterior (P), dorsal (D) and ventral (V), and medial (M) and lateral (L) sectors of the GPe (*blue*, *upper row*) and GPi (*red*, *lower row*). Data are expressed in millions (10^6^) of ChAT-immunostained axon varicosities per mm^3^ of tissue. ***P < 0.001, **P < 0.01 and *P < 0.05, by Wilcoxon signed-rank test

The total number of ChAT+ axon varicosities in the squirrel monkey pallidum, as estimated by means of an unbiased stereological approach, is of 2.8 ± 0.3 × 10^6^ in the GPi and 10.4 ± 1.6 × 10^6^ in the GPe. The mean volumes of the GPi and the GPe, as measured with Cavalieri’s method, are 10.7 ± 0.2 and 22.2 ± 0.5 mm^3^, respectively, leading to a significantly lower (*P* = 0.03) mean density of ChAT+ axon varicosities in the GPi (0.26 ± 0.03) compared to the GPe (0.47 ± 0.07), as expressed in million of axon varicosities per mm^3^ of tissue (Fig. 4). Interestingly, the anterior halves of both pallidal segments are more densely innervated than their posterior counterparts. The mean density of ChAT+ axon varicosities in the anterior GPi is 0.33 ± 0.02 compared to 0.21 ± 0.01 × 10^6^/mm^3^ in the posterior GPi (*P* < 0.0001), whereas the values for the anterior and posterior GPe are respectively 0.56 ± 0.05 and 0.38 ± 0.03 × 10^6^/mm^3^ (*P* < 0.0001). The ventral half of the pallidal complex is also more densely innervated by ChAT+ axon varicosities than its dorsal half, and this holds true for both pallidal segments. The mean density of the ChAT+ axon varicosities in the dorsal and ventral portions of the GPi are 0.25 ± 0.01 and 0.30 ± 0.02 × 10^6^/mm^3^ (*P* = 0.002), whereas the corresponding values for the GPe are 0.42 ± 0.04 and 0.60 ± 0.05 × 10^6^/mm^3^ (*P* = 0.001). The density of ChAT+ axon varicosities is similar in the lateral and medial sectors of the GPi, but significantly higher in the medial portion of the GPe compared to its lateral counterpart (0.53 ± 0.04 vs. 0.46 ± 0.04 × 10^6^/mm^3^, *P* = 0.04; Fig. 4).

### Number of ChAT-immunoreactive axon varicosities per pallidal neuron

The unbiased stereological approach used in the present study has allowed us to estimate the total neuronal population of the GPi at 36,984 ± 1,836 neurons compared to 87,073 ± 5,899 neurons for the GPe. Yet, because the GPi is much smaller (10.7 ± 0.2 mm^3^) than the GPe (22.2 ± 0.5 mm^3^), the neuronal density remains remarkably similar in the two pallidal segments. The GPi harbours 3.00 ± 0.13 thousands of neurons per mm^3^ of tissue vs. 3.62 ± 0.22 for the GPe. The evaluation of the neuronal density along the main axes of the two pallidal segments reveals that only the GPe displays a decreasing anteroposterior gradient, with 4.05 ± 0.13 × 10^3^ neurons/mm^3^ in its anterior half compared to 2.89 ± 0.07 in its posterior half (*P* < 0.0001). No significant variation is noted along the dorsoventral and mediolateral axes in both pallidal segments.

**Fig. 5:**
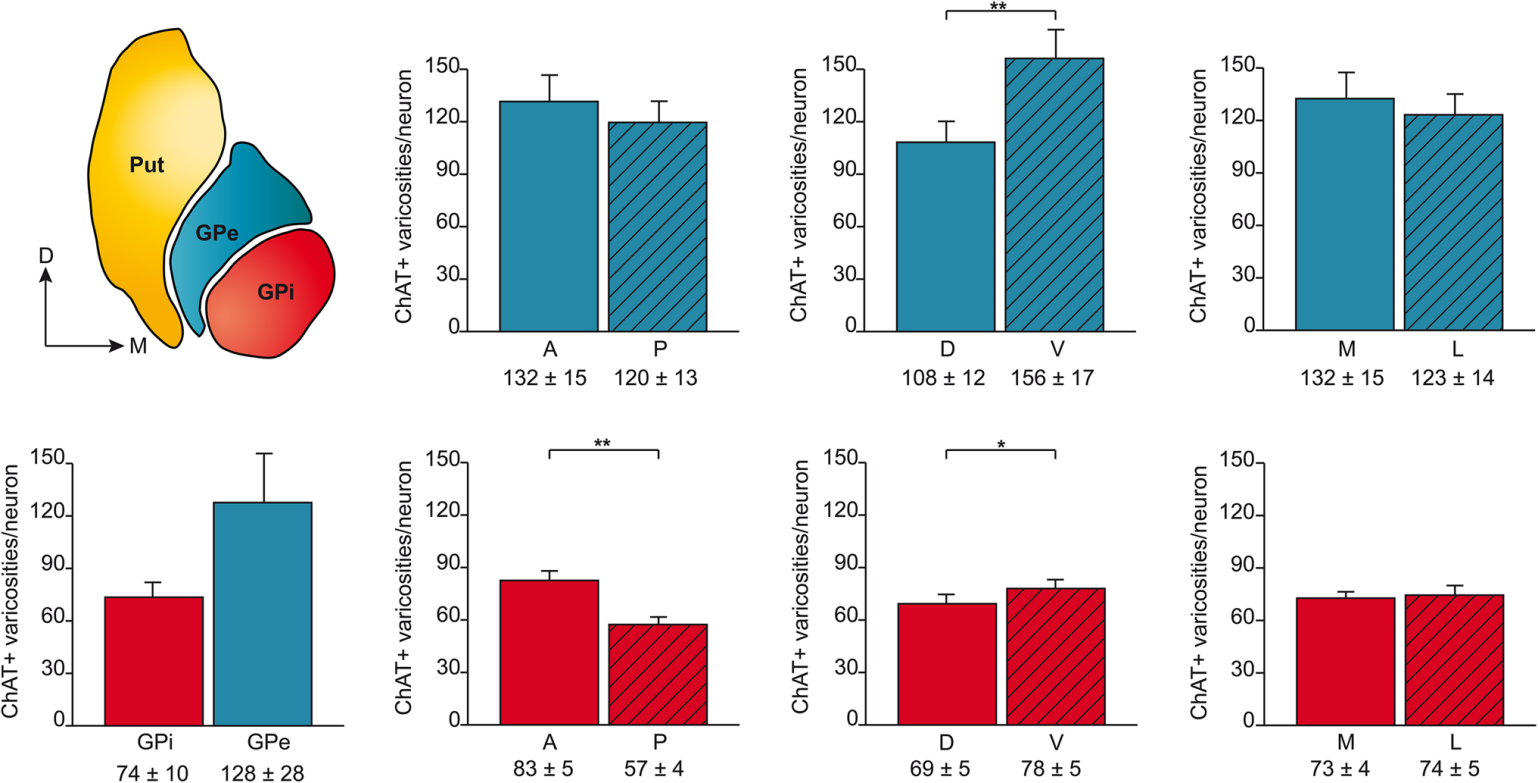
Average numbers of ChAT+ axon varicosities per neuron for the whole GPi (*red*) and the whole GPe (*blue*), as well as for the anterior and posterior, dorsal and ventral, and medial and lateral sectors of the GPe (*blue*, *upper histograms*) and GPi (*red*, *lower histograms*). **P < 0.01 and *P < 0.05, by Wilcoxon signed-rank test

The gathering of these data regarding the total numbers of pallidal neurons and ChAT+ axon varicosities in the two pallidal segments has allowed us to estimate the number of ChAT+ axon varicosities per single pallidal neuron (Fig. 5). The values obtained reveal that GPi neurons are less densely innervated (74 ± 10 varicosities/neuron) than GPe neurons (128 ± 28 ChAT+ varicosities/neuron). Some regional variations also exist within each pallidal segment in regards to the density of neuronal innervation. For example, neurons in the anterior half of the GPi are more densely innervated (83 ± 5 varicosities/neuron) than those in the posterior half (57 ± 4 varicosities/neuron) (*P* = 0.003), whereas an increasing gradient is detected along the dorsoventral axis of both GPi (69 ± 5 vs. 78 ± 5 varicosities/GPi neuron, *P* = 0.04) and GPe (108 ± 12 vs. 156 ± 17 varicosities/GPe neuron, *P* = 0.001). In contrast, the number of ChAT+ axon varicosities per pallidal neuron does not vary significantly along the lateromedial axis of both GPi and GPe.

### Ultrastructural features of ChAT-immunoreactive axon varicosities

**Fig. 6:**
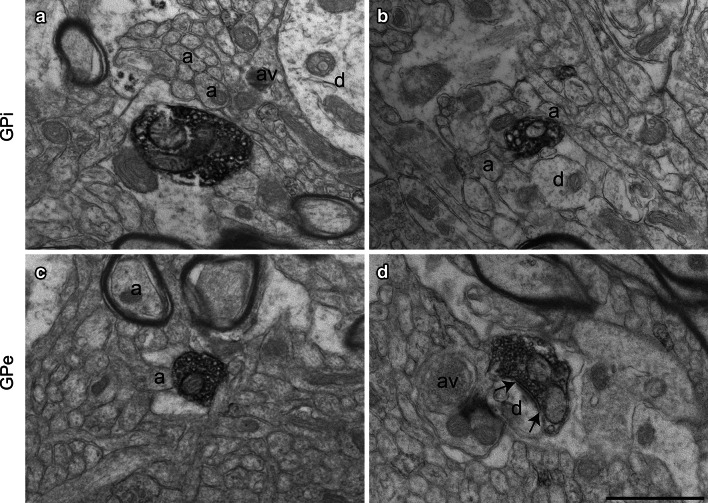
Examples of ChAT+ axon varicosities in the GPi (**a**, **b**) and the GPe (**c**, **d**) as visualized by electron microscopy after labeling with the immunoperoxidase-diaminobenzidine technique. ChAT+ axon varicosities observed in both GPi and GPe are usually surrounded by small unmyelinated or myelinated axons (**a**). Although none of the ChAT+ axon varicosities in **a**–**c** display an area of synaptic membrane specialization, a dendritic profile (**d**) is often seen in the surrounding microenvironment. In **d**, a ChAT+ axon varicosity is seen to make an asymmetrical synaptic contact (between *arrows*) with a small dendritic profile (**d**). *Scale bar* 1 µm

In both GPi and GPe, ChAT+ axon varicosities derive from unmyelinated axons, are generally ovoid, contain aggregated small and clear vesicles, and frequently display one or more mitochondria. Diaminobenzidine immunoprecipitate fills their axoplasm and typically line the plasma membrane and the outer surface of organelles (Fig. 6). Occasionally, ChAT-immunoreactive dendrites were observed in the GPi and GPe, some receiving a synaptic contact from unlabeled axon varicosities (Fig. 1f). These dendrites presumably belong to some of the few intrinsic ChAT+ neurons detected in the GPe under the confocal microscope (Fig. 1c).

The shape of the ChAT+ axon varicosities present in the two pallidal segments is similar, but these immunostained elements are significantly smaller than their unlabeled counterparts selected at random in the surrounding neuropil (Table 1). Statistically significant differences between ChAT+ and unlabeled axon varicosities in regards to their short axis, long axis and diameter [(long axis + short axis)/2] are noted in both GPi and GPe. The average diameter of ChAT+ varicosities is estimated at 0.48 ± 0.03 µm compared to 0.72 ± 0.03 µm for unlabeled profiles in the GPi (*P* = 0.0001, by Tukey’s post hoc test), whereas the corresponding values for the GPe are 0.56 ± 0.03 vs. 0.73 ± 0.02 µm (*P* = 0.005, by Tukey’s post hoc test). Differences in size between ChAT+ axon varicosities and unlabeled profiles can also be assessed by smaller cross-sectional area of ChAT+ varicosities in the GPi and the GPe (*F*_3,12_ = 20.10, *P* < 0.0001, by one-way ANOVA). The cross-sectional area of ChAT+ profiles observed in the GPi is 0.20 ± 0.02 µm^2^ compared to 0.44 ± 0.03 µm^2^ for unlabeled profiles (*P* = 0.0001, by Tukey’s post hoc test), and 0.27 ± 0.03 µm^2^ compared to 0.44 ± 0.03 for those observed in the GPe (*P* = 0.004, by Tukey’s post hoc test). Furthermore, the ChAT+ axon varicosities present in the GPi are overall smaller than those in the GPe, although this variation does not reach statistical significance.

**Table 1:** Morphometric features of ChAT-immunostained vs. randomly selected unlabeled axon varicosities in the monkey internal and external pallidum

	GPi (*n* = 4)		GPe (*n* = 4)	
	ChAT	Unlabeled	ChAT	Unlabeled
Number examined	197	182	213	193
Dimensions				
Short axis (μm)	0.35 ± 0.02***	0.55 ± 0.02	0.41 ± 0.03***	0.53 ± 0.01
Long axis (μm)	0.60 ± 0.04**	0.89 ± 0.04	0.71 ± 0.03**	0.92 ± 0.03
Aspect ratio	1.75 ± 0.07	1.69 ± 0.07	1.79 ± 0.08	1.81 ± 0.01
Diameter (μm)	0.48 ± 0.03***	0.72 ± 0.03	0.56 ± 0.03***	0.73 ± 0.02
Area (μm^2^)	0.20 ± 0.02***	0.44 ± 0.03	0.27 ± 0.03***	0.44 ± 0.03
% with mitochondria	48 ± 8	72 ± 6	64 ± 8	59 ± 5

Data are presented as mean ± SEM. The unlabeled profiles were selected at random from the same micrograph displaying ChAT+ profiles, as explained in “Materials and methods”

** *P* = 0.0002 and *** *P* = 0.0001 for ChAT vs. unlabeled

In both GPi and GPe, the proportion of ChAT+ axon varicosities that display a synaptic membrane specialization is small when compared to their unlabeled counterparts (*F*_3,12_ = 47.52, *P* < 0.0001, by one-way ANOVA). The synaptic incidence measured in single-thin section for ChAT+ profiles is 4.2 ± 0.3 % compared to 24.0 ± 3.0 % for unlabeled profiles in the GPi (*P* < 0.0001, by Tukey’s post hoc test) and 5 ± 1 % compared to 31 ± 2 % in the GPe (*P* < 0.0001, by Tukey’s post hoc test). By extrapolating the synaptic incidence measured from single-thin sections to the whole volume of varicosities with the stereological formula of Beaudet and Sotelo (1981), we estimate that only 12 ± 2 % of GPi ChAT+ and 17 ± 3 % of GPe ChAT+ axon varicosities are endowed with a synaptic junction, indicating that more than 80 % of the ChAT+ axon varicosities in the pallidum are devoid of any synaptic membrane specialization (Table 2). On the other hand, the same extrapolation made from unlabeled profiles in the GPi and GPe reveals a much higher prevalence of synaptic incidence (90 ± 10 and 125 ± 16 %, respectively), implying that many of these unlabeled axon varicosities may display more than one junctional complex. Differences in the synaptic incidence of ChAT+ axon varicosities and unlabeled counterparts reach statistical significance in both pallidal segments (*F*_3,12_ = 33.34, *P* < 0.0001; *P* < 0.001, by Tukey’s post hoc test for GPi and *P* < 0.0001, by Tukey’s post hoc test for GPe).

**Table 2:** Junctional characteristics of ChAT-immunostained vs. randomly selected unlabeled axon varicosities in the monkey internal and external pallidum

	GPi (*n* = 4)		GPe (*n* = 4)	
	ChAT	Unlabeled	ChAT	Unlabeled
Synaptic incidence (%)				
Single section	4.2 ± 0.3***	24 ± 3	5 ± 1***	31 ± 2
Whole volume	12 ± 2***	90 ± 10	17 ± 3***	125 ± 16
Length of synaptic junction (μm)	0.21 ± 0.04	0.25 ± 0.02	0.24 ± 0.02	0.24 ± 0.03
Junctions (%)				
Symmetrical	88 ± 13	83 ± 3	85 ± 9	85 ± 5
Asymmetrical	13 ± 13	17 ± 3	15 ± 9	15 ± 5

Data are from the same sectional varicosity profiles as in Table 1 (mean ± SEM). The varicosity profiles were classified as showing or not a synaptic junction according to the criteria described in “Materials and methods”. The synaptic incidence for the whole volume of varicosities was extrapolated from the formula of Beaudet and Sotelo (1981), using the long axis as diameter of profiles (Umbriaco et al. 1994)

*** *P* < 0.0001 for ChAT vs. unlabeled

The ChAT+ axon varicosities that display genuine synaptic membrane specialization contact exclusively dendritic profiles of pallidal neurons. The vast majority of junctional complex observed in the two pallidal segments are of the symmetrical type (88 % in the GPi and 85 % in the GPe, see Table 2). The postsynaptic targets of ChAT+ axon varicosities observed in the GPi and the GPe are smaller than that of unlabeled profiles, suggesting that synapses established by ChAT+ axon varicosities occur more distally on pallidal dendrites. Furthermore, ChAT+ postsynaptic targets observed in the GPi are smaller than those of the GPe, suggesting that ChAT+ synaptic contacts are established more proximally on GPe dendrites compared to GPi dendrites. The neuropil within which the ChAT+ axon varicosities are embedded in the GPi and GPe is chiefly enriched in small non-myelinated axons (70 % for the GPi and 68 % for the GPe), but also harbours astrocytes (14, 15 %), dendrites (11, 15 %) and myelinated axons (5, 7 %).

## Discussion

The present study has provided the first detailed description of the morphological organization of the cholinergic (ACh) innervation of the globus pallidus in primates. The light microscope quantitative estimates gathered in the squirrel monkey show that the ACh innervation is less dense in the GPi than in the GPe, a finding that shed a new light on the role of ACh in these two major components of the pallidal complex, which are involved in quite different aspects of basal ganglia functions. The electron microscopic results reveal that the vast majority of ACh axon terminals in the primate pallidum do not establish a synaptic contact, suggesting that ACh acts upon GPi and GPe neurons largely through volumic transmission. The functional significance of these novel findings will now be outlined in the light of the relevant literature data.

### Technical considerations

All experiments whose results are reported herein were undertaken under stringent conditions, allowing for specific and optimal visualization of ChAT-immunostained axon varicosities in the globus pallidus of squirrel monkeys. Complete penetration of immunoreagents through sections that were processed for light microscopy was confirmed by the z-depth histogram provided with StereoInvestigator analysis, ChAT+ axon varicosities being evenly distributed throughout the thickness of the optical disector. Thus, the counts of these immunostained varicosities within the delineated sectors and across the entire volume of the GPi and GPe led to a reliable estimate of ACh innervation that was based on a precise estimate of the number of axon varicosities per volumetric unit of tissue. It did not simply rely on the qualitative or quantitative assessment of the overall immunostaining intensity to which the presence of non-varicose axonal segments contributes.

### Origin and distribution of ACh axon varicosities in primate pallidum

The last two decades saw the publication of immunohistochemical studies containing information about the ACh innervation of various brain structures in rodents and primates (for review see Descarries and Parent 2014). Among the structures that have been examined with more or less details at the light microscopic level in primates are: (1) the cerebral cortex (Mesulam and Geula 1988; Mrzljak et al. 1995); (2) the claustrum (Sutoo et al. 1994); (3) the nucleus basalis of Meynert (Everitt et al. 1988; Mesulam and Geula 1988); (4) the amygdala (Sutoo et al. 1994); (5) the thalamus (Darvesh and Hopkins 2003); and (6) the hippocampus (Sutoo et al. 1994). Information is also available on ACh innervation of some basal ganglia components or related structures in primates, including: (1) the striatum (DiFiglia 1987; Everitt et al. 1988; Mesulam et al. 1992; Sutoo et al. 1994); (2) the subthalamic nucleus (Mesulam et al. 1992), (3) the substantia nigra (Mesulam et al. 1992; Lavoie and Parent 1994a); (4) the PPN (Everitt et al. 1988; Charara and Parent 1994; Lavoie and Parent 1994b); and (5) the globus pallidus (Mesulam et al. 1992; Sutoo et al. 1994; Charara and Parent 1994). These earlier studies have revealed, among other things, that virtually all basal ganglia nuclei receive an ACh innervation, the intensity of which varies significantly from one structure to the other. In human, for example, the density of the globus pallidus ACh innervation was found to be less than that of the striatum and subthalamic nucleus, but more intense than that of the substantia nigra pars compacta (Mesulam et al. 1992; Sutoo et al. 1994), a pattern similar to the one disclosed here for squirrel monkeys. However, the density of the ACh innervation of primate brain structures has never been studied with the stereological approach used in the present study, rendering any specific comparisons between our findings and those of these previous studies somewhat limited. Likewise, there exist no quantitative data on the ACh innervation of the pallidal complex of nonprimate species, precluding detailed inter-specific comparisons. Nevertheless, faithful quantitative estimates of the number of ACh axon varicosities have been obtained for various other brain regions in rats, such as the cerebral cortex (Mechawar et al. 2000), the hippocampus (Aznavour et al. 2002) and the thalamus (Parent and Descarries 2008). All these regions in rodents appear to be more densely innervated by ACh axons than the GPi and the GPe of squirrel monkeys, as determined in the present study. Whether such variations in numbers represent genuine brain region variations or interspecific (rodents-primates) differences remain to be ascertained.

The present demonstration of a significant ACh innervation of the pallidum in the squirrel monkey is in agreement with the results of our anterograde and retrograde labeling studies undertaken two decades ago in the same species (Charara and Parent 1994; Lavoie and Parent 1994c). However, some results of these early investigations are at odds with those of the present study. For example, the labeled fibers traced after anterograde tracers injection in the PPN were found to be more abundant in the GPi, particularly in its ventromedial sector, than in the GPe (Lavoie and Parent 1994c). In contrast, the present quantitative analysis reveals that the density of ACh axon varicosities as well as the number of ACh terminals per pallidal neuron are respectively 81 and 73 % lower in the GPi than in the GPe. Several factors may explain the discrepancy between the two sets of investigations. First of all, no attempt was made to establish the ACh nature of the labeled fibers in our original tract-tracing study. Second, the mapping of the anterogradely labeled fibers that we provided at that time included the presence of both thick and smooth fibers, which could have been fibers of passage en route to other basal ganglia nuclei, as well as thin and varicose axons that were more likely genuine terminal axonal segments (Lavoie and Parent 1994c). These various shortcomings were overcome in the present study by focussing specifically on the number of ACh axon varicosities that displayed ChAT immunoreactivity, which are considered as the major releasing site of ACh, rather than assessing the overall density of both smooth and varicose ACh axons. This way of doing has allowed us to provide a faithful estimate of the density of the ACh innervation in different pallidal sectors.

In contrast, much remains to be known about the exact cellular origin of the ACh axon terminals present at pallidal levels. For example, we still don’t know if the ACh innervation of the GPi and the GPe derives from a single PPN neuron endowed with a highly collateralized axon or from two distinct neuronal populations, each located in a specific sector of the PPN. The tracing of single axons from the rat PPN tends to support the first hypothesis (Mena-Segovia et al. 2008; Roš et al. 2010) and in agreement with such a view is the presence of thin, unmyelinated and highly collateralized axons displaying several axon varicosities in various sectors of the GPi and GPe, as detected in the present study. Single-axon tracing studies are obviously needed to confirm that the ACh innervation of the pallidum derives from collateralized axons of single PPN neurons in primates.

Besides the above-mentioned difference between the GPi and GPe, results obtained in human showed a decreasing anteroposterior gradient of ACh innervation (Mesulam et al. 1992), which concurs with our present findings of topographical variations in the density of the pallidal ACh innervation in squirrel monkeys. The significance of such regional variations is unknown, but it may reflect, at least in part, the functional organization imposed upon the pallidal complex by the massive striatal afferents (Parent 1990; François et al. 1994). Afferents from the so-called associative, sensorimotor and limbic striatal territories are thought to project principally to the anterodorsal, caudoventral and medial sectors of the pallidal complex in monkeys (for review see Parent and Hazrati 1995b). Hence, the numerous ACh axon varicosities present in the anterior and ventral sectors of both pallidal segments would be ideally placed to exert a strong modulation of incoming striatal inputs from the associative striatal territory, in addition to their modulation of pallidal neurons. However, the interpretation of the action of ACh on pallidal neurons in relation to the pallidal territory in which these neurons lie has some serious limitations. First, large zones of overlap exist between these three functional territories, which should not be considered as distinct functional entities with rigid boundaries (see Parent and Hazrati 1995b). Second, pallidal neurons in primates are endowed with dendrites that can reach more than 1 mm in length and these long dendrites are almost entirely covered by axon varicosities (Fox et al. 1974; DiFiglia et al. 1982). Such a morphological feature confers to pallidal neurons the capacity to integrate neuronal information originating from more than one functional pallidal domain.

### ACh neuronal cell bodies in primate pallidum

In a previous comparative study of the basal forebrain of rats, cats and rhesus monkeys, in which the putative ACh neurons were identified by their intense staining for the enzyme acetylcholinesterase (AChE), we showed that neurons of the nucleus basalis invade massively the globus pallidus (but not the entopeduncular nucleus) in rats and cats (Parent et al. 1979). At rostral pallidal levels, the AChE+ neurons present in the internal medullary lamina of rhesus monkeys surrounded nearly entirely the GPi, but did not invade the GPi itself (Parent et al. 1979). Large ChAT immunofluorescent neurons forming a ring around the GPi have also been clearly visualized in the present study (see above) and such neurons appear to be absent from the GPi itself. The distribution pattern of ACh neurons in primate basal forebrain, as detected in rhesus monkeys by using AChE histochemistry (Parent et al. 1979), has been confirmed by means of ChAT immunohistochemistry in both a previous study conducted in squirrel monkeys (Armonda and Carpenter 1991) and the present study. Indeed, in addition to numerous heterogeneously distributed ACh axon varicosities, the GPe is also endowed with some ACh nerve cell bodies and here we were able for the first time to quantify with unbiased methods the density of these large cell bodies in the GPe. Despite the fact that they represent less than 2 % of the GPe neuronal population, these intrapallidal ACh neurons have been seen to receive synaptic contacts on poorly ramified dendrites, but nothing is known on the arborization of their axon. We hypothesize that these neurons complement the ACh PPN innervation by providing local innervation of the GPe.

### Ultrastructural features of the ACh axon varicosities in primate pallidum

At the electron microscopic level, the ACh axon varicosities observed in the pallidal complex of squirrel monkeys are similar in size and shape to ACh varicosities that have been described previously in other parts of the primate brain (DiFiglia 1987; Frotscher et al. 1992; Mrzljak et al. 1995). In rats, ACh axon varicosities observed in the striatum (Contant et al. 1996), the thalamus (Parent and Descarries 2008) and the parietal cortex (Umbriaco et al. 1994) were reportedly smaller than their unlabeled counterparts, which concurs with our findings of significantly smaller ACh axon varicosities than unlabeled profiles in the GPi and GPe of squirrel monkeys. This finding suggests that, irrespective of their region of origin (brainstem, basal forebrain or striatum), the ACh influence within primate and rodent brains is exerted through numerous small axon terminals emitted by widely collateralized axons instead of by large terminals originating from poorly arborized fibers.

One of the most interesting findings of the present study is that the vast majority of ACh axon varicosities observed in the monkey GPi and GPe are devoid of synaptic contacts. Even more striking is the very high synaptic incidence of unlabeled axon varicosities selected at random on the same micrograph of ChAT-immunostained sections, suggesting that some of these unlabeled varicosities are endowed with more than one typical synaptic membrane specialization. A previous study of the ACh innervation of the prefrontal cortex in adult rhesus monkey has reported a similar low synaptic incidence (Mrzljak et al. 1995). This investigation also showed that among 100 serially sectioned ChAT-immunostained axon varicosities, only 44 % made synaptic contact. Using two samples of human temporal lobe removed at surgery (Smiley et al. 1997), it was reported that 67 % of 42 ACh axon varicosities were endowed with synapses. In the rat, low synaptic incidences were also observed in the striatum (Contant et al. 1996; Aznavour et al. 2003), the hippocampus (Aznavour et al. 2005), and the dorsal geniculate and reticular thalamic nuclei (Parent and Descarries 2008). In contrast, virtually all ACh varicosities detected in the parafascicular thalamic nucleus of the rat were of the synaptic type (Parent and Descarries 2008; reviewed in Descarries and Parent 2014). Furthermore, in accordance with observations made in the cerebral cortex of the rat (Umbriaco et al. 1994; Mechawar et al. 2002) and monkey (Mrzljak et al. 1995), as well as in the rat striatum (Contant et al. 1996; Aznavour et al. 2003), the rare ACh synaptic contacts that were observed and reported in the present study are mostly of the symmetrical type.

Our results provide morphological evidence of a diffuse transmission of ACh in addition to a synaptic transmission in the primate globus pallidus, as it has been suggested in various nonpallidal brain areas for ACh and for monoaminergic systems (see Descarries and Mechawar 2008). In contrast to what has been described in other brain areas of nonprimate species, the density of ACh axon varicosities observed in the monkey pallidum is rather low and may not be sufficient to maintain a significant ambient level of ACh that could permanently exist in the extracellular space (Descarries et al. 1997). Nevertheless, the firing patterns of pallidal neurons may certainly be modulated by local fluctuations of ACh leading to activation of muscarinic or nicotinic receptors located either on their somatodendritic domains or on specific pallidal afferents (Wada et al. 1989; Bernard et al. 1992; Quik et al. 2000; Yan et al. 2001). Indeed, electrophoretic application of ACh in the pallidum of anesthetized rats has been found to increase as well as decrease pallidal neuronal activity (Lénárd et al. 1995). Moreover, experiments conducted with slice preparations has shown that activation of nicotinic (Kayadjanian et al. 1994) and muscarinic (Kayadjanian et al. 1997) receptors leads to a transient increase of GABA concentration in the rat pallidum, probably through the activation of pre-synaptic ACh receptors located on striatopallidal axons. In accordance with this hypothesis is our observation that ACh terminals do not engage in axo-axonic synaptic contact, suggesting that the activation of such pre-synaptic receptors occurs through volume transmission, whereas direct modulation of pallidal neurons is exerted through both synaptic and diffuse transmission of ACh. These findings are particularly relevant to the understanding of the pathophysiology of various neurodegenerative conditions, particularly Parkinson’s disease, where the interplay between the striatopallidal GABAergic inhibitory projections and the subthalamopallidal glutamatergic excitatory projections is markedly perturbed.

### Comparison between the ACh and the 5-HT innervations of primate pallidum

Similarly to ACh, serotonin (5-HT) is a major neuromodulator originating in the brainstem and giving rise to a dense and heterogeneous innervation of the primate globus pallidus (Eid et al. 2013). Both neurotransmitters have the ability to interfere with firing patterns of pallidal neurons and with their afferent projections, based on the location of their specific receptors. Direct comparisons between the ACh pallidal innervation reported here and the data on 5-HT pallidal innervation gathered in the same animals using the exact same stereological parameters (Eid et al. 2013) reveal interesting differences and similarities regarding morphological features between these two brainstem pallidal afferents. Indeed, our former stereological investigation using the serotonin transporter (SERT) as a marker of 5-HT axons and axon varicosities, revealed that the GPi and GPe harbour 0.57 and 0.60 × 10^6^ SERT+ axon varicosities/mm^3^, respectively (Eid et al. 2013). These values indicate that the density of the 5-HT innervation is about 54 % higher than that of the ACh innervation in GPi and 22 % higher in the GPe. Furthermore, the density of the 5-HT innervation was rather similar in the GPi and GPe (Eid et al. 2013), whereas the latter pallidal segment receives a more intense ACh innervation than the former segment. These findings indicate that the brainstem ascending 5-HT axons arborize rather uniformly within the two pallidal segments, in contrast to the ACh ascending system, which targets preferentially the GPe. This conclusion is further supported by estimates of the number of 5-HT axon varicosities per neuron that are similar in the GPi and GPe (Eid et al. 2013), and the values for ACh that are considerably lower in the GPi than in the GPe. This finding suggests that ACh innervation might have a stronger influence on GPe neurons, a key integrator of the basal ganglia, than on GPi neurons, the main output structure of the basal ganglia, whereas the effect of the 5-HT innervation would be about the same on GPi and GPe neurons. Obviously, the exact locations of 5-HT and ACh inputs on pallidal neurons as well as location of different types of 5-HT and ACh receptors on pallidal dendrites have to be taken into account to better assess the relative strength of ACh and 5-HT inputs.

Our previous study of the 5-HT innervation of the primate pallidum has revealed that the 5-HT axon varicosities are smaller than their unlabeled congeners, as it is also the case for ACh axon varicosities. The 5-HT axon varicosities also resemble the ACh axon varicosities by the fact that only a minority of them display a synaptic specialization, which occurs exclusively on pallidal dendrites of smaller caliber than those targeted by unlabeled profiles. Furthermore, no 5-HT axo-axonic synapses were detected, in agreement with the absence of ACh axo-axonic synapses. Altogether, these findings suggest that both the 5-HT and ACh brainstem ascending systems exert their well-established modulatory action upon various pallidal afferents mainly through diffuse transmission, whereas their direct control of pallidal neurons results from both volumic and synaptic release of their transmitter, the latter phenomenon occurring predominantly at distal dendritic levels.
